# The effect of endogenously released glucose, insulin, glucagon-like peptide 1, ghrelin on cardiac output, heart rate, stroke volume, and blood pressure

**DOI:** 10.1186/1476-7120-9-43

**Published:** 2011-12-29

**Authors:** Joanna Hlebowicz, Sandra Lindstedt, Ola Björgell, Magnus Dencker

**Affiliations:** 1 Center for Emergency, Department of Cardiology, Skåne University Hospital, Malmö, Lund University, Sweden; 2 Department of Cardiothoracic Surgery, Skåne University Hospital, Lund, Lund University, Sweden; 3 Department of Radiology, Skåne University Hospital, Malmö, Lund University, Sweden; 4 Department of Clinical Physiology and Nuclear Medicine, Skåne University Hospital, Malmö, Lund University, Sweden

## Abstract

**Background:**

Ingestion of a meal increases the blood flow to the gastrointestinal organs and affects the heart rate (HR), blood pressure and cardiac output (CO), although the mechanisms are not known. The aim of this study was to evaluate the effect of endogenously released glucose, insulin, glucagon-like peptide 1 (GLP-1), ghrelin on CO, HR, stroke volume (SV), and blood pressure.

**Methods:**

Eleven healthy men and twelve healthy women ((mean ± SEM) aged: 26 ± 0.2 y; body mass index: 21.8 ± 0.1 kg/m^2^)) were included in this study. The CO, HR, SV, systolic and diastolic blood pressure, antral area, gastric emptying rate, and glucose, insulin, GLP-1 and ghrelin levels were measured.

**Results:**

The CO and SV at 30 min were significantly higher, and the diastolic blood pressure was significantly lower, than the fasting in both men and women (*P *< 0.05). In men, significant correlations were found between GLP-1 level at 30 min and SV at 30 min (*P *= 0.015, r = 0.946), and between ghrelin levels and HR (*P *= 0.013, r = 0.951) at 110 min. Significant correlations were also found between the change in glucose level at 30 min and the change in systolic blood pressure (*P *= 0.021, r = -0.681), and the change in SV (*P *= 0.008, r = -0.748) relative to the fasting in men. The insulin 0-30 min AUC was significantly correlated to the CO 0-30 min AUC (*P *= 0.002, r = 0.814) in men. Significant correlations were also found between the 0-120 min ghrelin and HR AUCs (*P *= 0.007, r = 0.966) in men. No statistically significant correlations were seen in women.

**Conclusions:**

Physiological changes in the levels of glucose, insulin, GLP-1 and ghrelin may influence the activity of the heart and the blood pressure. There may also be gender-related differences in the haemodynamic responses to postprandial changes in hormone levels. The results of this study show that subjects should not eat immediately prior to, or during, the evaluation of cardiovascular interventions as postprandial affects may affect the results, leading to erroneous interpretation of the cardiovascular effects of the primary intervention.

**Trial registration number:**

NCT01027507

## Background

Several kinds of postprandial cardiovascular changes have been reported in the literature. The postprandial blood flow in the superior mesenteric artery (SMA) seems to be approximately double the fasting value, and initiates an increase in cardiac output (CO) [1]. Postprandial CO increase has been suggested to result from increases in the heart rate (HR) and stroke volume (SV). The postprandial blood flow in the SMA increases simultaneously with the gradual increase in the CO, reaching a maximum 30 to 60 min after eating. A large meal increases CO more, and for a longer time, than a small meal [2]. However, the composition of the meal seems to be less important in this respect [3]. The ingestion of food has also been shown to decrease the diastolic blood pressure [4,5].

The autonomic innervation of the stomach and the heart is divided into the parasympathetic and sympathetic systems. The parasympathetic innervation is controlled by the vagus nerve, whose cell bodies are found in the brainstem. The vagus nerve consists of 20% efferent fibres and 80% afferent sensory fibres, which transmit information to the brain [6]. The vagus nerve mediates the adaptive relaxation of the proximal stomach, the fundic-antral co-ordination (by controlled delivery of the food from the fundus to the antrum) and the peristaltic contractions of the distal stomach after a meal [7]. Sympathetic stimulation increases the HR (positive chronotropy), inotropy and conduction velocity (positive dromotropy), whereas parasympathetic stimulation of the heart has the opposite effects [6]. Changes in HR, heart rate variability (HRV), and blood pressure are some of the factors that may reflect the balance between the sympathetic and parasympathetic nervous systems. It appears that neural signals are less likely to be responsible for the increase in CO [8]. In order to investigate denervated hearts, Waaler at al. investigated subjects with transplanted hearts and found that the CO, HR and SV increased in a similar way to that of control subjects [4,8]. It is still not known what causes the changes in the pumping activity of the heart postprandially. Our hypothesis was that the intake of food would change the activity of the heart due to postprandial changes in the antral area, or levels of glucose, insulin, GLP-1 and ghrelin. It is known that insulin has positive chronotropic and inotropic effects on the heart [9], and the hormone glucagon-like peptide 1 (GLP-1) has been shown to improve left ventricular function [10,11]. The hormone ghrelin has been shown to increase CO and stroke volume (SV) [12-15]. Postprandial haemodynamic changes have been shown to resemble the effects of vasodilator drugs [16,17]. The evaluation of cardiovascular interventions postprandially may affect the results, leading to erroneous interpretation of the cardiovascular effects of the primary intervention. The aim of this study was to evaluate the effect of endogenously released glucose, insulin, GLP-1, ghrelin on CO, HR, stroke volume (SV), and blood pressure.

## Methods

Twenty-three healthy subjects (11 men, 12 women; (mean ± SEM) aged: 26 ± 0.2 y (range: 18-33 y); body mass index: 21.8 ± 0.1 kg/m^2 ^(range: 17.0-25.9 kg/m^2^)) without symptoms or a history of gastrointestinal disease, abdominal surgery or diabetes mellitus, were included in this observational study. The mean waist:hip ratio of the women was 0.74 ± 0.02 and of the men 0.87 ± 0.01. The subjects had no connective tissue disease or cerebrovascular or endocrine disease, and none was taking any medication, except four women who were taking oral contraceptives. Three men were snuff users and one was a smoker, while two of the women were snuff users. All subjects were recruited from the population of southern Sweden.

All subjects gave their written informed consent. The study was approved by the Ethics Committee of Lund University, and performed according to the Helsinki Declaration. The study started on 13 January 2009 and ended on 18 September 2009. The trial is registered in the US National Library of Medicine with the trial registration number NCT01027507.

The subjects were examined between 7.30 and 11.00 a.m. after an 8-h fast. Smoking and snuff-taking were prohibited 8 h prior to and during the examination. The fasting blood glucose concentration of each subject was measured on the day of the examination to ensure that it was normal (≤ 7.0 mmol/L). If the subjects reported gastrointestinal symptoms (diarrhoea or constipation) on the day of the study, the examination was postponed.

The test meal consisted of 300 g rice pudding (AXA Goda Gröten Risgrynsgröt; Lantmännen AXA, Järna, Sweden). The total caloric value of the meal was 330 kcal: 10% from protein (9 g), 58% from carbohydrates (48 g) and 32% from fat (12 g). The meal was ingested within 5 minutes. The gastric emptying rate (GER) was estimated using an ultrasound method described previously [18]. The sonographic examination was performed with a 3.5-MHz abdominal transducer and an imaging system ((Acusone Sequioa 512, Mountain View, CA), (Siemens Elegra, Siemens Medical Solutions, Mountain View, CA)). Measurements were made 15 and 90 min after the meal had been consumed, and the degree of gastric emptying was expressed as the percentage change in the antral cross-sectional area between these two measurements. At each examination, the longitudinal and anteroposterior diameters were measured three times, and the mean values were used to calculate the cross-sectional area of the gastric antrum. The GER (%) was calculated using the following equation:

$$\text{GER }=\;\left(\text{1}-\left(\text{Antrum area 9}0\text{ min}∕\text{Antrum area 15 min}\right)\right)\;\times \text{ 1}00$$

Measurements of the gastric antrum were performed by the same radiologist. The abdominal aorta and the left lobe of the liver were used as internal landmarks in the measurements on the gastric antrum. The subjects were examined in the supine position, and were not allowed to sit up between examinations.

**Figure 1:**
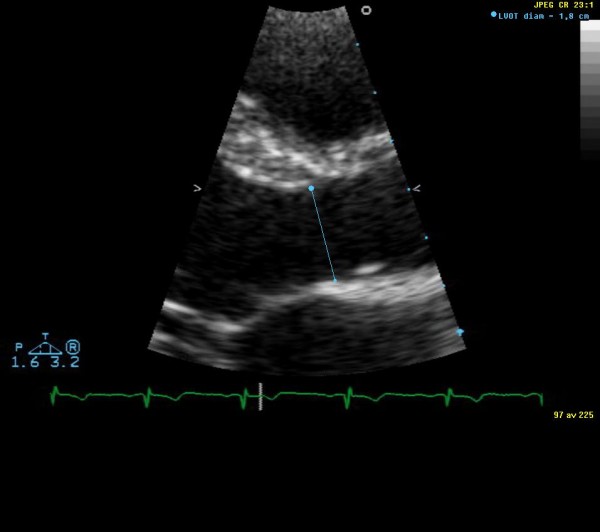
**Representative image of measurement of the left ventricular outflow tract diameter**.

**Figure 2:**
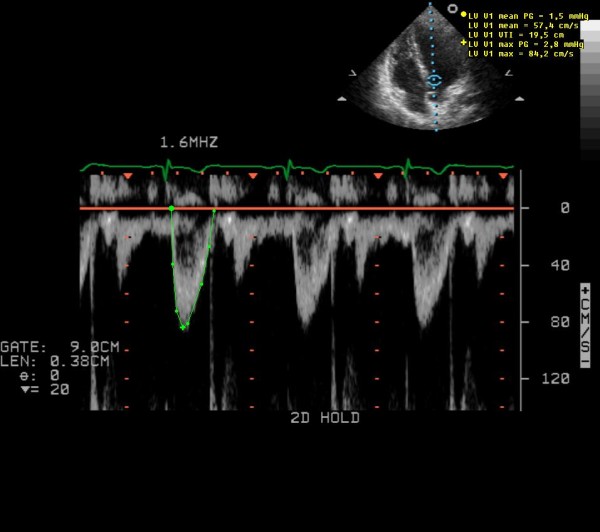
**Representative measurement of the left ventricular outflow tract velocity-time integral**.

Blood pressure was measured in the supine position using a conventional (mechanical) sphygmomanometer with an aneroid manometer and a stethoscope. Systolic pressure (first phase) was identified by the first of the continuous Korotkoff sounds. Diastolic pressure was identified as the moment the Korotkoff sounds disappeared (fifth phase). Transthoracic echocardiography examinations were performed with a Sonos 5500 ultrasound system (Philips, Andover, MA, USA) in the left lateral position, after 15 minutes' rest. A single observer performed all the echocardiography measurements. The left ventricular SV was measured, and the left ventricular CO was calculated according to current guidelines [19]. The Doppler method was used SV = D^2 ^× 0.785 × VTI, where VTI = left ventricular outflow tract velocity-time integral and D = left ventricular outflow tract diameter. CO was calculated as SV × Heart rate. A separate intra-observer variability study concluded low variability for CO and SV measurement (< 5%). Figure 1 is a representative image of measurement of the left ventricular outflow tract diameter. Figure 2 is a representative measurement of left ventricular outflow tract velocity-time integral. Blood pressure and echocardiogram examinations were performed by the same observer before the meal (0 min) and 30, and 110 min after the start of the meal.

Venous blood samples were taken before and 15, 30, 45, 60, 90, and 120 after the start of the meal to measure glucose, insulin, GLP-1 and ghrelin levels. Dipeptidyl peptidase-IV inhibitor was added to the low pressure blood test bottles before collection of blood for GLP-1. We were not able to collect venous blood samples for the measurement of GLP-1 and ghrelin levels in 6 men and 3 women for technical reasons'. Blood glucose concentrations were measured with the HemoCue glucose system (HemoCue AB, Ängelholm, Sweden). The precision of this system is better than 0.3 SD between 0 mmol/L and 22.2 mmol/L. Insulin concentrations were measured using an immunoassay with an alkaline phosphatase conjugate (Access Ultrasensitive Insulin, Beckman-Coulter AB, Bromma, Sweden). The sensitivity of the insulin immunoassay is 0.03 mUnit/L (mU/L), and the intra-assay coefficient of variation was below 10% between 0.03 mU min/L and 300 mU/L.

Plasma ghrelin concentrations were determined using a radioimmunoassay kit (GHRT-89k) manufactured by Linco Research (St. Charles, Missouri, USA), which measures total ghrelin (intact as well as desoctanoylated). The sensitivity obtained was 100 pg/mL, and the intra-assay coefficients of variation were below 10%. Quality controls were always within acceptable limits. GLP-1 concentration in plasma was measured after extraction of plasma with 70% ethanol (vol/vol, final concentration). The plasma concentrations of GLP-1 were measured against standards of synthetic GLP-1 7-36 amide using antiserum code no. 89390 [20], which is specific to the amidated C-terminus of GLP-1, and therefore does not react with GLP-1-containing peptides from the pancreas. The results of the assay accurately reflect the rate of secretion of GLP-1 as the assay measures the sum of intact GLP-1 and the primary metabolite, GLP-1 9-36 amide, into which GLP-1 is rapidly converted [21]. The sensitivity was below 1 pmol/L, the intra-assay coefficient of variation was below 6% at 20 pmol/L, and the recovery of the standard added to plasma before extraction was approximately 100% when corrected for losses inherent in the plasma extraction procedure.

### Statistical analysis

All analyses were performed for men and women separately. Results are given as mean values and SEM, unless otherwise stated. The total areas under the curves (AUCs) were calculated for blood glucose, insulin, GLP-1, ghrelin, CO, HR, SV, and systolic and diastolic blood pressure in each subject using GraphPad Prism software (version 4; GraphPad, San Diego, CA, USA). All other statistical calculations were performed in SPSS for Windows (SPSS, Version 14.0, 2005; Chicago, IL, USA). Changes in the values of blood glucose, insulin, GLP-1 and ghrelin levels were calculated as the difference between the levels before the meal (fasting value) and 30 and 120 min after the end of the meal. Changes in CO, HR, SV, and systolic and diastolic blood pressure were calculated as the difference between levels before the meal (fasting value) and 30 and 110 min after the end of the meal. To determine whether the meal affected a given parameter, the fasting value was compared with the values 30 min and 120 min after the meal, using the Wilcoxon t-test. The values at 30 min and 120 min postprandially were also compared using the Wilcoxon t-test. The significance in differences between a given parameter in women and men was determined using the Mann-Whitney U test. Possible correlations between blood pressure, CO, HR, SV and levels of blood glucose, plasma insulin, GLP-1, ghrelin, or antral area were analysed with the Pearson correlation. The level of statistical significance was set at *P *< 0.025 after Bonferroni correction.

## Results

### Postprandial glucose, insulin, GLP-1 and ghrelin responses

**Figure 3:**
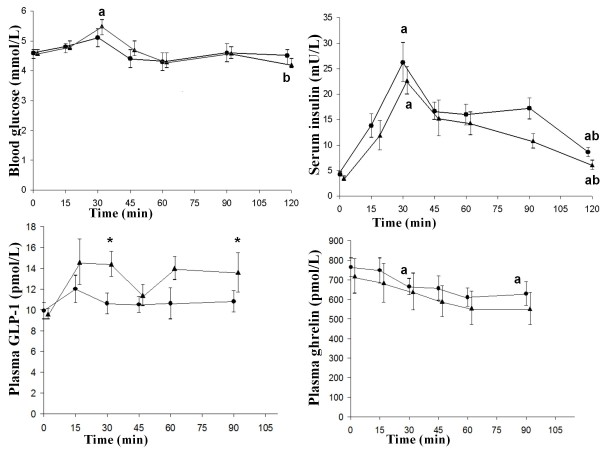
**The mean (± SEM) glucose, insulin, GLP-1 and ghrelin concentrations in twelve healthy women (**•**) and eleven healthy men (▲), after the ingestion of a meal consisting of rice pudding**. The letter a indicates a statistically significant difference compared with the fasting value, according to the Wilcoxon t-test (p < 0.05). The letter b indicates statistically a significant difference compared with the value at 30 min, according to the Wilcoxon U test (p < 0.05). *A significant difference was also found between the men and the women, according to the Mann-Whitney U test (*P *< 0.05).

The glucose level at 30 min was significantly higher than the fasting in (*P *= 0.023) and at 120 min in men (*P *= 0.004) (Figure 3). The insulin levels at 30 min and 120 min were significantly higher than the fasting in both men (*P *= 0.003, *P *= 0.05) and women (*P *= 0.003, *P *= 0.003). The insulin level at 30 min was significantly higher than at 120 min in both men (*P *= 0.003) and women (*P *= 0.006). The insulin level at 120 min (*P *= 0.019) and insulin AUC 0-120 min (*P *= 0.019) were significantly higher in the women than men (Figure 3). Changes, relative to fasting values in GLP-1 at 30 min and 120 min, were 4.8 ± 0.8, and 4.0 ± 0.3 pmol/l in men, respectively. Changes, relative to fasting values in GLP-1 at 30 min and 120 min, were 1.8 ± 0.7, and 0.4 ± 0.9 pmol/l in women, respectively. The change in GLP levels at 30 min and 120 min (*P *= 0.018 and *P *= 0.019) were significantly higher in men than women. The ghrelin levels at 30 min and 120 min were significantly lower than the fasting in women (both *P *= 0.008) (Figure 3).

### Cardiovascular parameters

**Figure 4:**
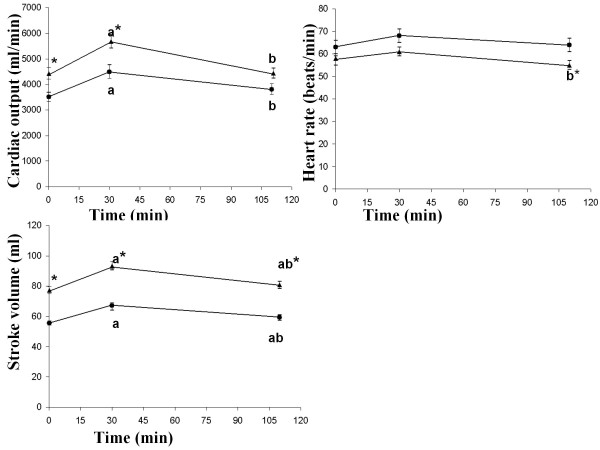
**The mean (± SEM) cardiac output (CO), heart rate (HR), and stroke volume (SV) in twelve healthy women (**•**) and eleven healthy men (▲), after the ingestion of a meal consisting of rice pudding**. The letter a indicates a significant difference in response in the postprandial phase compared to fasting, calculated using the Wilcoxon t-test (*P *< 0.05). The letter b indicates statistically a significant difference compared with the value at 30 min, according to the Wilcoxon U test (p < 0.05). *A significant difference was also found between the men and the women, according to the Mann-Whitney U test (*P *< 0.05).

The CO at 30 min was significantly higher than the fasting in both women (*P *= 0.005) and men (*P *= 0.003). The CO at 30 min was also significantly higher than at 110 min in both women and men (both *P *= 0.003). The CO at fasting and at 30 min was significantly higher in men than in women (*P *= 0.006 and *P *= 0.013, respectively) (Figure 4). The HR at 110 min was significantly lower than at 30 min in men (*P *= 0.018). The HR at 110 min was significantly lower in men than women (*P *= 0.019) (Figure 4). The SV at 30 min was significantly higher than the fasting in both women (*P *= 0.002) and men (*P *= 0.003). The SV at 110 min was significantly higher than the fasting in women (*P *= 0.016). The SV at 30 min was significantly higher than at 110 min in both women (*P *= 0.008) and men (*P *= 0.005). The SV at fasting, at 30 min and at 110 min was significantly higher in men than women (*P *= 0.000) (Figure 4). The AUCs for CO 0-30 min were 151622 ± 6781 in men, and 59967 ± 3214 mL in women. The AUCs for CO 0-120 min were 556323 ± 22442 in men, and 454132 ± 25116 mL in women. The AUCs for SV 0-30 min were 2562 ± 248 in men, and 914 ± 28 mL min in women. The AUCs for CO 0-30 min, CO 0-110 min, SV 0-30 min were significantly higher in men than women (*P *= 0.000, *P *= 0.019 and *P *= 0.000, respectively). The AUCs for HR 0-30 min were 1771 ± 59 in men, and 979 ± 39 beats in women. The AUCs for HR 0-120 min were 6408 ± 202 in men, and 7237 ± 313 beats in women. The AUCs for HR 0-30 min (*P *= 0.000) and HR 0-110 min (*P *= 0.000) were significantly lower in men than women.

**Figure 5:**
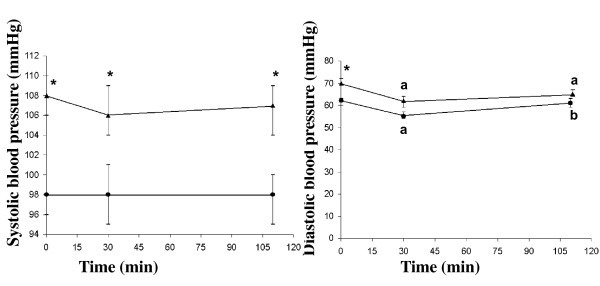
**The mean (± SEM) systolic and diastolic blood pressure in twelve healthy women (**•**) and eleven healthy men (▲), after the ingestion of a meal consisting of rice pudding**. "a" indicates a significant difference in response in the postprandial phase compared to fasting, calculated using the Wilcoxon t-test (*P *< 0.05). The letter b indicates statistically a significant difference compared with the value at 30 min, according to the Wilcoxon U test (p < 0.05). *A significant difference was also found between the men and the women, according to the Mann-Whitney U test (*P *< 0.05).

The systolic blood pressure at fasting, and at 110 min was significantly higher in the men than the women (*P *= 0.004, *P *= 0.013, respectively) (Figure 5). The diastolic blood pressure at 30 min was significantly lower than the fasting in both women (*P *= 0.003) and men (*P *= 0.012). The diastolic blood pressure at 30 min was significantly lower than at 110 min in the women (*P *= 0.006). The diastolic blood pressure at fasting was significantly lower in the women than in the men (*P *= 0.001) (Figure 5). The AUCs systolic blood pressure 0-30 min were 3220 ± 39 in men, and 2939 ± 68 mmHg min in women. The AUCs systolic blood pressure 0-120 min were 11723 ± 225 in men, and 10769 ± 254 mmHg min in women. The AUCs diastolic blood pressure 0-30 min were 1985 ± 50 in men, and 1759 ± 39 mmHg min in women. The AUCs for systolic blood pressure: 0-30 min and 0-110 min (*P *= 0.003 and *P *= 0.013), and diastolic blood pressure: 0-30 min (*P *= 0.002) were significantly higher in the men than the women.

### Correlations

Statistically significant correlations were only found in men. Significant correlations were found between GLP-1 level at 30 min and SV at 30 min (*P *= 0.015, r = 0.946). There were also significant correlations between ghrelin levels and HR (*P *= 0.013, r = 0.951) at 110 min.

Significant correlations were also found between the change in glucose level at 30 min and the change in systolic blood pressure (*P *= 0.021, r = -0.681), and the change in SV (*P *= 0.008, r = -0.748) relative to the fasting. The insulin 0-30 min AUC was significantly correlated to the CO 0-30 min AUC (*P *= 0.002, r = 0.814). Significant correlations were also found between the 0-120 min ghrelin and HR AUCs (*P *= 0.007, r = 0.966)

## Discussion

The aim of this study was to evaluate the effect of endogenously released glucose, insulin, glucagon-like peptide 1, ghrelin on CO, HR, SV, and blood pressure. Our hypothesis was that the intake of food would change the activity of the heart due to postprandial changes in the antral area, and levels of glucose, insulin, GLP-1 and ghrelin. We were able to partly verify this hypothesis. The results of the study show that postprandial CO and SV increased in both men and women. The ingestion of food decreased the diastolic blood pressure, without affecting the systolic blood pressure. In men, the postprandial changes in glucose levels were positively correlated to systolic blood pressure, the GLP-1 levels to SV, the insulin levels to CO and the ghrelin level to HR. To our best knowledge, this is the first study to examine at the effect of, endogenously released insulin, glucagon-like peptide 1, ghrelin levels on CO, SV, HR, systolic and diastolic blood pressure in both men and women.

In our study, the ingestion of food led to a decrease in the diastolic blood pressure, without affecting the systolic blood pressure. Previous studies have shown that gastric distension influences blood pressure, probably due to activation of the gastrovascular reflex in patients with autonomic failure [22-24], and in older healthy subjects [25]. However, the systolic blood pressure was not affected in healthy adolescents [24]. Gastric distension has been shown to be attenuated in the elderly [26]. In the present study, the systolic blood pressure was not affected postprandially. The reason why we did not observe any correlation between blood pressure and antral area may be because we evaluated only young healthy subjects with a normal gastric emptying rate and normal autonomic innervation of the stomach and heart. The gastric distension was not quantified by measurements of the intragastric volume.

In a previous study on two men and two women, Waaler reported postprandial increases in CO and SV, which were explained by an increase in HR [1]. In a slightly larger study, on seven men and one woman, Kelbaek found an increase in postprandial CO, HR and SV [27]. Our results suggest slightly greater early postprandial HR, but no statistically significant differences were found. A gradual increase in CO 30 min to 1 h after eating, and a relationship between the CO and meal size has been suggested to reflect the postprandial changes in the gastrointestinal tract [1]. The increases in CO and splanchnic blood flow seem to be synchronous [1], indicating a relation between postprandial changes in the splanchnic blood flow and changes in the activity of the heart. The vagus nerve mediates the fundic-antral co-ordination by controlled delivery of the food from the fundus to the antrum [7]. The postprandial antral area has been observed to have a maximal value at 15 min in healthy subjects, thereafter decreasing, and reaching a plateau value close to the fasting antral area 75 to 90 min after meal ingestion [18]. In our study, the antral cross-sectional area was not correlated to the postprandial changes in SV, CO or blood pressure. In subjects with transplanted (i.e. denervated) hearts, Waaler found that CO, HR and SV increased in a similar way to those of control subjects postprandially [4,8]. Therefore, it does not seem likely that neural signals are responsible for the increase in CO.

In the present study, it was found that postprandial changes in glucose and GLP-1 levels were positively correlated to SV, and that insulin levels were positively correlated to CO in men. Intravenous insulin administration has previously been reported to increase the CO before any significant decrease in glucose levels was seen, in six healthy male subjects [16]. A dose-dependent physiological increase in CO has been reported when insulin was infused during a hyperinsulinaemic euglycaemic clamp in men [28]. Infusion of insulin thus causes vascular effects similar to the food-induced haemodynamic changes observed in our study. There seems to be an effect of meal size on postprandial increase in CO [1]. Also, physiological hyperinsulinemia affects autonomic control and reduces diastolic blood pressure, increase HR and CO [29]. Our current understanding of the effects of GLP-1 on the heart is mainly based on studies using exogenous GLP-1, whereas little is known about the effects of endogenously released GLP-1. In a recent study, the infusion of GLP-1 was reported to have no effect on the left ventricular ejection fraction (LVEF) in 20 patients with heart failure [30]. However, another study showed that a 5-week infusion of GLP-1 improved the LVEF in twelve patients with heart failure (7 men, 5 women) [11]. A 72-hour infusion of GLP-1 in ten patients (7 men, 3 women) with left ventricular dysfunction following acute myocardial infarction and reperfusion has also been reported to improve the LVEF [10]. It has been suggested that central GLP-receptor stimulation reduces the parasympathetic vagal modulation of the heart [31]. It has also been suggested that GLP-1 has inotropic effects [32,33], and may improve endothelial function [34,35]. Treatment with GLP-1 receptor agonist in patients with type 2 diabetes has been associated with a lower risk of CVD events and hospitalizations that treatment with other glucose-lowering therapies [36]. Also, treatment with GLP-1 receptor agonist in subjects with type 2 diabetes has been associated with lower systolic blood pressure without effects on HR [37].

We observed significantly greater changes in GLP-1 levels, compared with fasting values, in the men than in the women in this study. The reason for these differences is not known, but could be related to the fact that we did not observe any correlations between postprandial GLP levels and haemodynamic responses in women. We observed no differences in ghrelin levels between the genders, but the ghrelin levels were correlated to the HR in the men in this study. It has been suggested that ghrelin suppresses sympathetic activity [38]. Intravenous and subcutaneous ghrelin injections have been reported to increase CO and improve cardiac contractility in healthy subjects [12]. Treatment with ghrelin has also been shown to decrease systemic vascular resistance and increase CO, cardiac index, and SV in patients with heart failure [13].

Postprandial haemodynamic responses in men and women were analysed separately in this study as there are known gender differences in heart function [39-45]. There are also gender-related differences in cardiovascular regulation. Females have a higher HR and lower HRV than men [46-48]. Reports on these autonomic responses are not always in agreement, for example, it has been claimed that women show higher [49] and lower [48] parasympathetic effects, and lower sympathetic activity [47], and that men have higher parasympathetic activity [46]. Overnight fasting has been suggested to be associated with an increase in parasympathetic activity that is counteracted by eating breakfast [50]. The response has been reported to differ in that men show greater parasympathetic activity than women [50]. Thus, it appears that there are also gender-related differences in the haemodynamic responses to postprandial changes in hormone levels. However, the gender differences might be due to differences in the level of insulin and GLP-1. The difference the hemodynamic response to glucose and ghrelin remain unclear. There are also known gender differences in cardiovascular diseases and this may be related to sex hormones, estrogen and testosterone [51].

The present study had some limitations: the small sample size of healthy young subjects and the fact that it was not possible to perform the echocardiography or gastric ultrasound examinations at the same time. However, the measurements of the gastric antrum and the heart were performed by the same radiologist. The results of the echocardiography examinations were stored digitally and analysed at a later date in an attempt to avoid bias.

## Conclusions

This study shows that postprandial CO and SV increase in both men and women. Ingestion of food decreased the diastolic blood pressure without affecting the systolic blood pressure or heart rate in healthy subjects. Physiological changes in the levels of glucose, insulin, GLP-1 and ghrelin may influence the activity of the heart and the blood pressure. There may also be gender-related differences in the haemodynamic responses to postprandial changes in hormone levels. Subjects undergoing evaluation of cardiovascular interventions should therefore refrain from eating before or during such examinations, as postprandial affects may affect the results, leading to erroneous interpretation of the cardiovascular effects of the primary intervention.

## Abbreviations

AUCs: areas under the curves; CO: in cardiac output; GER: gastric emptying rate; GLP-1: glucagon-like peptide 1; HR: heart rate; HRV: heart rate variability; LVEF: left ventricular ejection fraction; SMA: superior mesenteric artery; SV: stroke volume.

## Competing interests

The authors declare that they have no competing interests.

## Authors' contributions

The authors' contributions were as follows: JH and MD designed the study; JH was responsible for recruiting the subjects. OB performed the ultrasound examinations; MD performed the echocardiographic examinations; JH, SL, and MD carried out the statistical calculations. JH and MD wrote the first draft of the manuscript, and SL and OB made critical revisions of the manuscript. All authors read and approved the final manuscript.
